# The Multifunctional Ca^2+^/Calmodulin-Dependent Kinase IIδ (CaMKIIδ) Regulates Arteriogenesis in a Mouse Model of Flow-Mediated Remodeling

**DOI:** 10.1371/journal.pone.0071550

**Published:** 2013-08-08

**Authors:** Jason A. Scott, Paula J. Klutho, Ramzi El Accaoui, Emily Nguyen, Ashlee N. Venema, Litao Xie, Shuxia Jiang, Megan Dibbern, Sabrina Scroggins, Anand M. Prasad, Elisabeth D. Luczak, Melissa K. Davis, Weiwei Li, Xiaoqun Guan, Johannes Backs, Annette J. Schlueter, Robert M. Weiss, Francis J. Miller, Mark E. Anderson, Isabella M. Grumbach

**Affiliations:** 1 Department of Medicine, Carver College of Medicine, University of Iowa, Iowa City, Iowa, United States of America; 2 Department of Molecular Physiology and Biophysics, Carver College of Medicine, University of Iowa, Iowa City, Iowa, United States of America; 3 Department of Pathology, Carver College of Medicine, University of Iowa, Iowa City, Iowa, United States of America; 4 Iowa City VA Medical Center, Iowa City, Iowa, United States of America; 5 Department of Cardiology, University of Heidelberg, Heidelberg, Germany; Goethe University, Germany

## Abstract

**Objective:**

Sustained hemodynamic stress mediated by high blood flow promotes arteriogenesis, the outward remodeling of existing arteries. Here, we examined whether Ca^2+^/calmodulin-dependent kinase II (CaMKII) regulates arteriogenesis.

**Methods and Results:**

Ligation of the left common carotid led to an increase in vessel diameter and perimeter of internal and external elastic lamina in the contralateral, right common carotid. Deletion of CaMKIIδ (CaMKIIδ−/−) abolished this outward remodeling. Carotid ligation increased CaMKII expression and was associated with oxidative activation of CaMKII in the adventitia and endothelium. Remodeling was abrogated in a knock-in model in which oxidative activation of CaMKII is abolished. Early after ligation, matrix metalloproteinase 9 (MMP9) was robustly expressed in the adventitia of right carotid arteries of WT but not CaMKIIδ−/− mice. MMP9 mainly colocalized with adventitial macrophages. In contrast, we did not observe an effect of CaMKIIδ deficiency on other proposed mediators of arteriogenesis such as expression of adhesion molecules or smooth muscle proliferation. Transplantation of WT bone marrow into CaMKIIδ−/− mice normalized flow-mediated remodeling.

**Conclusion:**

CaMKIIδ is activated by oxidation under high blood flow conditions and is required for flow-mediated remodeling through a mechanism that includes increased MMP9 expression in bone marrow-derived cells invading the arterial wall.

## Introduction

Occlusive vascular disease is highly prevalent among older patients and can lead to limb loss and stroke [1]. The current treatment options of endarterectomy, bypass surgery and balloon angioplasty are limited by significant perioperative morbidity and mortality in an elderly patient population. An alternative strategy is to stimulate arteriogenesis, a process defined as outward remodeling of preexisting arteries induced by increased blood flow after occlusion of a collateral artery [2]. Thus, developing new non-invasive approaches to increase arteriogenesis may decrease the high morbidity and mortality associated with occlusive vascular disease.

All steps of arteriogenesis are likely coordinated through a temporal pattern of cytokine, chemokine, growth factor, and protease expression [3]. Mechanistically, the remodeling process in arteriogenesis is initiated by elevated flow in the collateral arteries, which increases endothelial surface shear stress, followed by an increase in radial stress [3]. The collateral vessel increases in diameter in the first weeks after occlusion until the stress is normalized. Arteriogenesis requires the interaction of endothelial and smooth muscle cells in the vascular wall with bone marrow cells of the monocyte/macrophage lineage [4]. In response to increased shear stress, the endothelium increases the expression of adhesion molecules [5] and releases cytokines that attract circulating monocytes [6]–[8], which adhere to and invade the collateral vessel wall. Matrix metalloproteases (MMPs) [9], [10], mainly secreted by infiltrating macrophages, are activated in the vascular wall and the pericollateral space and degrade the extracellular matrix surrounding the growing vessel, thereby producing a space into which the collateral arterial wall can expand. What remains largely unknown are the upstream regulators of flow-mediated remodeling.

Increased shear stress results in rises in intracellular calcium ([Ca^2+^]_i_) [11] and reactive oxygen species (ROS) [12], both of which activate the multifunctional Ca^2+^/calmodulin dependent kinase II (CaMKII). CaMKII is activated after conformational reordering that follows the binding of Ca^2+^-bound calmodulin (Ca^2+^/CaM) to the regulatory domain. CaMKII can become constitutively active, independent of Ca^2+^/CaM binding, by autophosphorylation of Thr 287 or oxidation of Met 281,282 in the regulatory domain [11]. These post-translational modifications lead to sustained CaMKII activity even after cellular Ca^2+^ levels decline to baseline values. CaMKII is robustly expressed in endothelium, vascular smooth muscle cells (VSMC) [13] and monocytes [14], [15]. We and others have demonstrated that CaMKII promotes VSMC proliferation and migration [16]–[19], two mechanisms that have been implicated in arteriogenesis [20], [21]. Recently, we reported that CaMKII regulates the expression of the matrix metalloproteinase MMP9 [17], a major regulator of arteriogenesis [9]. CaMKII function in the endothelium is currently incompletely defined, but nascent evidence suggests that it may regulate endothelial permeability [22], [23], a necessary event for monocyte invasion into the vascular wall.

We hypothesized that CaMKII mediates flow-mediated remodeling. Using an established *in vivo* model in which ligation of the left common carotid artery results in outward remodeling of the right carotid [4], [9], [24], our data demonstrate a pivotal role for CaMKII in arteriogenesis. CaMKII expression and activity were strongly increased in the right carotid artery. Outward remodeling was significantly decreased in *in vivo* models of CaMKIIδ deletion or inhibition of oxidative CaMKIIδ activation. While we did not observe any reduction in expression of adhesion or pro-inflammatory markers in CaMKIIδ−/− mice, we detected a significant decrease in adventitial MMP9 that colocalized with the extracellular space and macrophages. The transplantation of WT bone marrow into CaMKIIδ−/− mice recovered flow-mediated remodeling to the level seen in WT mice. In summary, these data demonstrate that CaMKII regulates arteriogenesis likely via induction of adventitial MMP9 expression in macrophages.

## Methods

### Reagents

The following antibodies were used in this study: anti-α-smooth muscle actin, anti-IL-6, anti-GAPDH (Santa Cruz Biotechnology), anti-Ki67 (BD Biosciences), anti-CD 68, anti-CD3, anti-CD31, anti-MMP9 (Abcam), anti-CD177 (Bioss), anti-CD45, anti-F4/80 (R&D), anti-p-CaMKII (Cell Signaling Technology), anti-VCAM1 and anti-MCP-1 (Abbiotec). Fluorescein-labeled Griffonia simplifolia lectin was purchased from Vector Labs. The generation of the anti-CaMKII [16] and anti-ox-Met 281/282 CaMKII antibodies [25] was described previously.

### Mice

CaMKIIδ−/− mice were kindly provided by Dr. Eric N. Olson, University of Texas, Dallas, TX and CaMKIIδ M281,282V mice were provided by Dr. Mark E. Anderson, University of Iowa. In CaMKIIδ M281,282V mice, the methionine residues 281 282 are mutated to valine [25], [26].

This study was carried out in strict accordance with the recommendations in the Guide for the Care and Use of Laboratory Animals of the National Institutes of Health. The protocol was approved by the Institutional Animal Care and Use Committee of the University of Iowa (IACUC# 0905097 and 1111234). The compliance with the protocol was verified by a representative of the University of Iowa Office of Animal Resources who observed procedures.

The animals were housed in a state-of-the-art facility that is accredited by AAALAC. All surgery was performed under anesthesia with ketamine and xylazine, and all efforts were made to minimize suffering. The methods of euthanasia were consistent with the recommendations by the American Veterinary Association.

### Carotid Injury Model

Ten- to 12-week-old wild-type C57Bl/6, CaMKIIδ−/− and CaMKIIδ M281,282V mice were anesthetized with ketamine and xylazine (2 mg and 0.3 mg, respectively, intraperitoneally (IP)). The left common carotid artery was ligated through a midline neck incision [27]. At 7, 14 or 28 days after injury of the left common carotid artery, all animals were anesthetized and perfused at physiological pressure with PBS followed by 4% paraformaldehyde for 3 minutes. The right carotid arteries were excised, paraffin- or cryo-embedded and subjected to immunohistochemical analysis as described below.

### Carotid Ultrasound and Blood Pressure Measurement

Carotid ultrasounds were performed using a Vevo 2100 System (VisualSonics) [28]. The mice were lightly sedated with midazolam (0.15 mg subcutaneously). 2-D cross-sectional images of the neck were acquired first using a 40-MHz linear-array probe. After identifying the common carotid arteries, longitudinal and cross-sectional views of the vessels were obtained. Pulsatile flow was confirmed using color and pulsed wave Doppler. The carotid luminal diameters were measured offline during systole over ten cardiac cycles.

Systolic blood pressure was measured using a noninvasive tail-cuff method (ADInstruments) as previously described [13] in trained mice on day 13 and 27 after administration of an identical regimen of midazolam as described for ultrasound.

### Histology and Immunohistochemistry

For analyses of flow-mediated remodeling, 5 µm sections were collected on Superfrost Plus slides. For morphometric assessment, the outermost (EEL) and innermost (IEL) elastic lamina was traced in 10 right carotid arteries 14 and 28 days after left carotid artery ligation using NIH Image J (20 sections per mouse). Some sections were H&E-stained prior to morphometric analysis.

Cell proliferation was assessed 14 and 28 days after carotid ligation by immunohistochemistry for the thymidine analogue 5-bromo-2′-deoxyuridine (BrdU; 2 mg per injection). BrdU was injected IP at 12 hours and 1 hour before the mice were sacrificed [16]. Right carotid arteries were sectioned as described for morphometric analyses and BrdU incorporation detected by immunofluorescence (Invitrogen). Data were normalized to the total number of intimal and medial VSMC, which were counted after nuclear staining with TO-PRO-3 (Invitrogen).

For immunostaining, 5 µm right carotid artery sections were subjected to heat-mediated antigen retrieval using 0.01 M citrate buffer and permeabilized in 1% Triton X-100 for 10 min. Sections were washed in PBS and then non-specific binding was blocked using a M.O.M. kit (Vector Labs) for 1 hr followed by incubation in anti-α-smooth muscle actin antibody (1∶200) for 30 min at room temperature. After washing in PBS for 30 min at room temperature, sections were preincubated in 5% goat serum for 30 min and then incubated with anti-MMP9 (1∶100), anti-p-CaMKII (1∶100), anti-ox-CaMKII (1∶100), anti-CaMKII (1∶100), anti-VCAM-1, anti-CD45, anti-CD177, anti-Mac-3 (1∶50), anti-CD3 or anti-F4/40 (1∶10) overnight at 4°C. The primary antibodies were detected with AlexaFluor 488- or 568-conjugated secondary antibodies (Invitrogen) or by 3,3′-Diaminobenzidine (DAB) histochemistry. Sections were counterstained with TO-PROIII, Syto16 or Vectashield containing DAPI (Vector Labs) to visualize nuclei. Images were captured with Zeiss LSM 510 META Laser confocal microscope. Densitometry for different antigens was performed using NIH Image J.

### Isolation and Culture of Macrophages

Bone marrow was isolated from two mouse femurs and tibias in ice-cold, sterile PBS. The bone marrow cells were plated and incubated in bone marrow macrophage (BMM) media (RPMI-1640 buffered with 25 mM Hepes and supplemented with 100 U/ml penicillin/streptomycin, 15% fetal calf serum, and 20% conditioned media from L929 fibroblasts). After 2 days, bone marrow cells of the non-monocyte/macrophage lineage, which adhered to the flask, were discarded and only monocytes/macrophages cells in the supernatant were used for experiments. Following 5 additional days of maturation in BMM media, the BMMs were treated with 1 µg/mL lipopolysaccharide (LPS) for 6 hr.

### qrtPCR

Total RNA was isolated using the RNeasy Kit (Qiagen) following the manufacturer’s recommendations. Preparation of the RNA included digestion with proteinase K and DNase I to eliminate possible genomic DNA contamination. cDNA was prepared from 1 µg total RNA using iScript cDNA Synthesis Kit (Bio-Rad) and random nanomer primers. Expression was quantified using an iQ Lightcycler instrument (Bio-Rad) with SYBR green dye and normalized to acidic ribosomal phosphoprotein (ARP) rRNA [13].

### CaMKII Activity Assays

Right carotid arteries were explanted on day 14 after left carotid ligation and 5 arteries pooled for protein isolation. CaMKII activity assays were performed using 5 µg protein as described previously [29].

### MMP9 Activity Assay

Right carotid arteries were explanted on day 7 after left carotid ligation and 5 carotid arteries were pooled for protein isolation. Active MMP9 in carotid homogenates was detected with the SensoLyte Plus 520 MMP-9 assay as recommended by the manufacturer. 0.5 µg protein was assayed in duplicate. The MMP9 activator 4-aminophenylmercuric acetate was added to the standards but not to the samples in order to specifically detect active MMP9 in the lysates.

### Bone Marrow Transplantation

C57Bl/6 wild type donors were euthanized at 8 weeks of age. Bone marrow was isolated from femurs by aspiration using a 23G needle and collected in sterile PBS. Bone fragments were removed by filtration. Bone marrow mononuclear cells were isolated using density gradient centrifugation with Ficoll/Lite-LM (Atlanta Biologicals). Red blood cells were lysed by incubation in Tris-NH_4_Cl for 5 min at 37°C. Cells were subsequently washed and resuspended in PBS. Recipient WT and CaMKIIδ−/− mice were irradiated with 1100 cGy (500+600 cGy at a 4 h interval). After a 4 h recovery, 1×10^6^ donor BM cells (0.2 mL of cell suspension) were injected into the retro-orbital plexus. The recipient mice recovered for 8 weeks to allow for full hematopoietic reconstitution. At 8 weeks post-transplantation, the recipient mice then underwent left carotid ligation. The right carotids were collected after 4 weeks for morphometric analysis. This time point was chosen based on our data in WT and CaMKIIδ−/− mice that demonstrated a significant difference in carotid size at this time point.

### Statistical Analysis

Data are shown as mean± SE unless noted otherwise. The SigmaPlot statistical package was used for the quantitative analyses of parameters such as intima-medial lesion area and intimal-medial SMC number (ANOVA with appropriate corrections for post-hoc analysis for multiple group comparisons and Student t test for comparison of two groups). A probability value <0.05 was considered significant. All quantitative assays were performed in duplicate or triplicate and repeated three times. The sample sizes per time point for the morphometry experiments were calculated to detect a 1.2-fold difference with a standard deviation of 20% with a two-sided α = 5% and a β-error of 50% (n = 5).

## Results

### Deletion of CaMKIIδ Prevents Flow-mediated Remodeling

**Figure 1:**
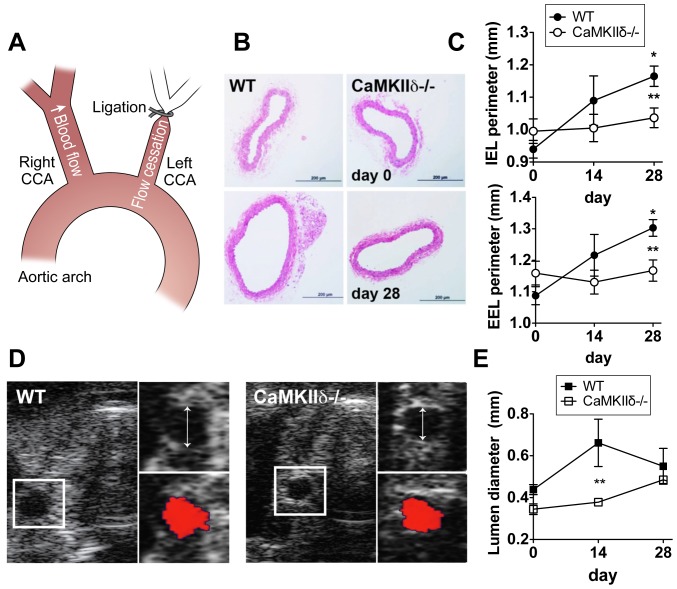
CaMKIIδ is required for flow-mediated remodeling. (A) Diagram of experimental approach. Arteriogenesis is induced in the right common carotid artery (CCA) after left CCA ligation. (B) Representative H&E-stained right carotid arteries of WT and CaMKIIδ−/− mice at baseline (day 0) and day 28 post-left carotid ligation. Scale bar = 200 µm. (C) Quantification of the perimeter of the internal (IEL) and external elastic lamina (EEL) (n = 6 for day 0 and n = 10 for days 14 and 28). (D) Ultrasound cross-sectional images of the right common carotid artery 28 days after left carotid ligation. The insets demonstrate color Doppler flow in the right carotid. No flow was detected in the left common carotid. (E) Quantification of the anterior-posterior diameter of right common carotid arteries of WT and CaMKIIδ−/− mice (n = 10 per genotype, experiments are independent of (B) and (C)). *p<0.05 compared to baseline; **p<0.05 compared to WT.

We investigated the role of CaMKIIδ in arteriogenesis using a carotid ligation model. Ligation of the left common carotid induces arteriogenesis in the contralateral right common carotid as a compensatory response [4], [9], [24] (Figure 1A). The left carotid arteries of CaMKIIδ−/− mice and wild type littermate controls (WT) were ligated and the degree of outward remodeling in the right carotid 14 and 28 days post-ligation was assessed by morphometric methods. In H&E-stained cross-sections of WT right carotid arteries, the external (EEL) and internal (IEL) elastic laminae perimeters increased significantly by 28 days post-ligation relative to baseline measurements in WT mice (Figure 1B, C). In contrast, the perimeters in CaMKIIδ−/− carotid arteries at 28 days were not statistically different from baseline. Baseline EEL or IEL perimeters were similar between genotypes. We did not detect any difference in blood pressure between genotypes that might explain the decrease in outward remodeling in CaMKIIδ−/− mice (Figure S1A in File S1).

The blunted arteriogenesis in CaMKIIδ−/− mice seen in histological sections was independently confirmed *in vivo* by ultrasound analysis (Figure 1D, E). Consistent with histological measurements, we detected an increase in the systolic luminal diameter in WT mice on day 28 post-ligation relative to baseline diameters but not in CaMKIIδ−/− mice. Increased blood flow post-ligation in the right artery was confirmed by Doppler ultrasound (Figure S1B in File S1). Taken together, *ex vivo* and *in vivo* measurements indicate that deficiency of CaMKIIδ prevents compensatory arteriogenesis. We observed an earlier increase in luminal size using ultrasound analysis by day 14, in comparison to morphometric measurements, that may be a reflection of an initially increased arterial distensibility in systole, whereas the structural remodeling as assessed by morphometry may reach its peak at a later time point.

### CaMKII Expression and Activity Increase during Arteriogenesis

**Figure 2:**
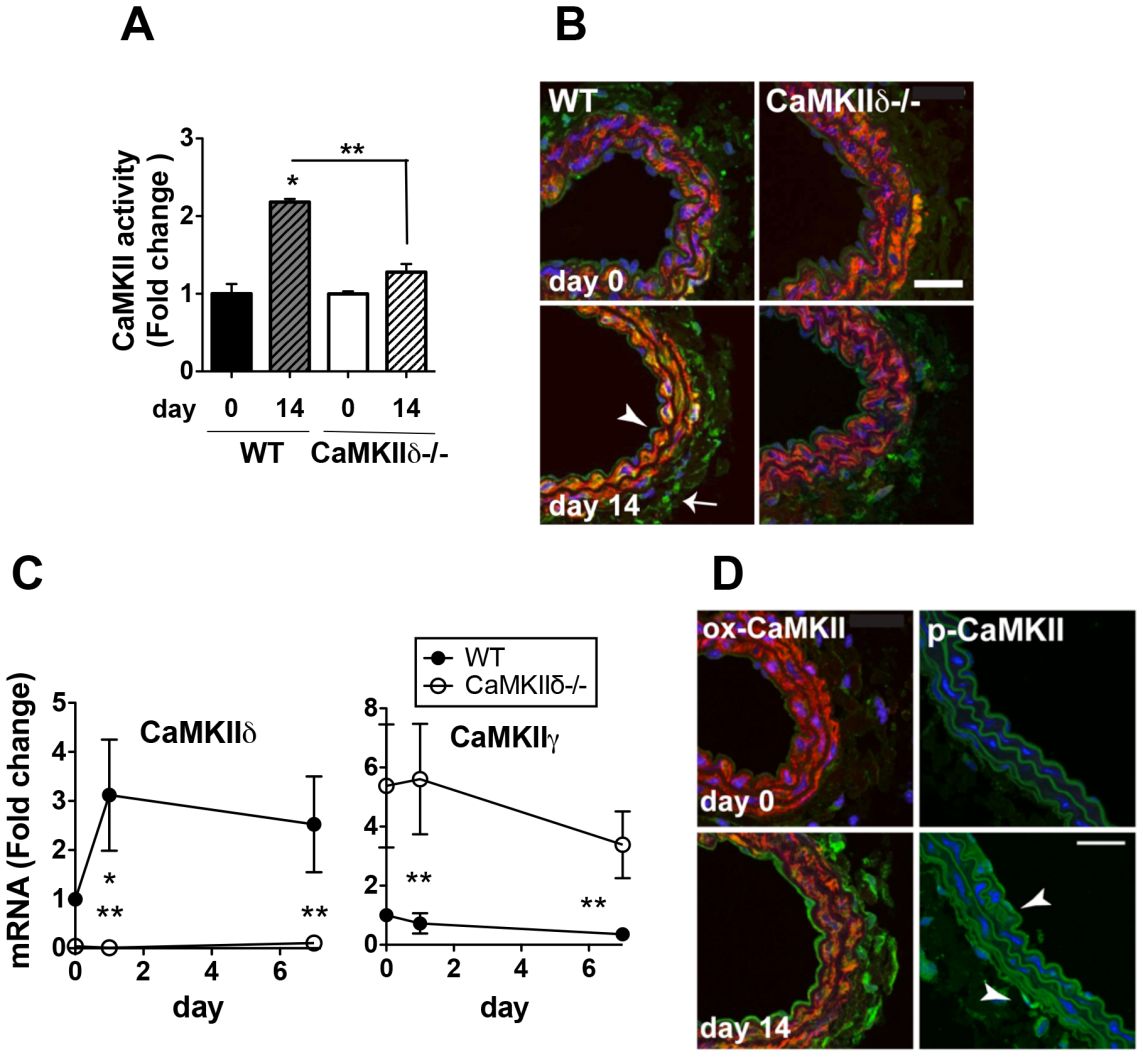
CaMKII is upregulated and activated in arteriogenesis. (A) CaMKII activity in right carotid arteries from WT and CaMKIIδ−/− mice 14 days after left carotid ligation. (B) Immunolabeling for total CaMKII (green; SM–actin red; nuclei blue) in WT and CaMKIIδ−/− right carotid artery sections on day 14 after ligation. Arrow, adventitia; arrowhead, endothelium. (C) Fold change in mRNA expression of CaMKIIδ and γ in right carotids isolated from WT and CaMKIIδ−/− mice by quantitative RT-PCR. (D) Immunolabeling for oxidized (ox-CaMKII, green, left panel) and phosphorylated CaMKII (p-CaMKII, green, right panel) in WT right carotid artery sections (SM–actin red; nuclei blue). Arrowheads indicate single cells with p-CaMKII labeling. Scale bar = 30 µm.

Next, we tested whether vascular remodeling induced by an increase in blood flow alters CaMKII expression or activity. At baseline in WT and CaMKIIδ−/− mice, the right carotid arteries had similarly low levels of CaMKII activity and expression (Figure 2A, B). Flow-mediated remodeling in WT carotid arteries increased CaMKII protein expression and activity. CaMKII expression was primarily elevated in WT endothelium and adventitia (Figure 2B, Figure S2A in File S1). In carotid arteries of CaMKIIδ−/− mice, the lack of outward remodeling was concomitant with blunted CaMKII expression and activity. CaMKIIδ mRNA expression increased in WT carotid arteries after ligation (Figure 2C). We also assessed the expression of CaMKIIγ, the other CaMKII isoform prevalent in the cardiovascular system. Interestingly, CaMKIIγ mRNA levels did not increase in response to increased flow in either genotype. CaMKIIγ mRNA in CaMKIIδ−/− arteries was higher relative to WT, consistent with a compensatory increase in this isoform with CaMKIIδ deficiency (Figure 2C). These findings support a view that the increase in CaMKII activity and protein expression under increased flow is mainly due to an increase in CaMKIIδ.

### Inhibition of Oxidative CaMKII Activation Prevents Flow-mediated Remodeling

CaMKII activation via oxidation mechanistically contributes to myocardial pathology [25], [26], [30], but its role in vascular physiology is incompletely understood [17]. The strong increase in.

ROS in the vascular wall in this model of arteriogenesis [31] suggests that CaMKII may be activated by oxidation. We next evaluated whether CaMKII is activated by oxidation or autophosphorylation. During arteriogenesis, oxidation of CaMKII was substantially increased in endothelial and adventitial cells from WT carotids (Figure 2D, Figure S2B in File S1), whereas autophosphorylated CaMKII was barely detectable in the arterial wall (Figure 2D). Moreover, an increase of peroxynitrite has been reported in the vascular wall in models of flow-mediated remodeling [32], [33]. In *in vitro* experiments, peroxynitrite directly activated CaMKII (Figure S3 in File S1). The activation was abrogated when the oxidative activation site of CaMKII at Met 281,282 was mutated to Val.

**Figure 3:**
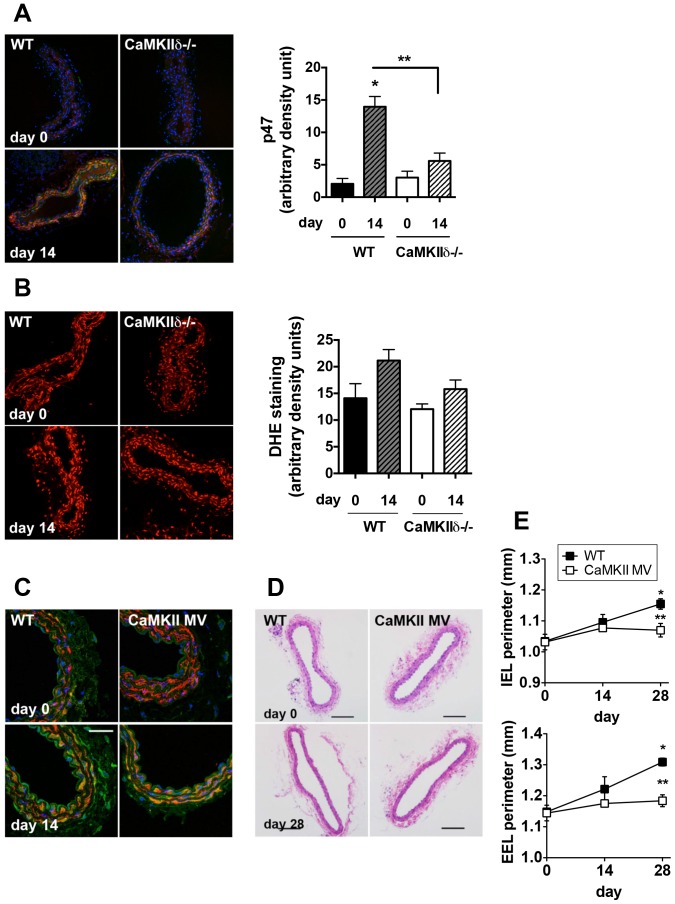
ROS and oxidative activation of CaMKII in the right carotid artery after left carotid ligation. (A) NADPH oxidase subunit p47 expression in WT and CaMKIIδ−/− right carotid arteries at baseline and on day 14 after left carotid ligation. (B) ROS production in the vascular wall of WT and CaMKIIδ−/− right carotid arteries at baseline and on day 14 after left carotid ligation. (C) Immunolabeling for ox-CaMKII (green; SM-actin, red; nuclei, blue) in WT and CaMKII MV carotid artery sections at baseline and 14 days post-ligation. (D) Representative H&E-stained right carotid arteries of WT and CaMKII MV mice. (E) Quantification of the perimeter of the IEL and EEL (n = 6 for day 0 and n = 10 for day 14 and 28). *p<0.05 compared to baseline; **p<0.05 compared to WT. Scale bar = 30 µm in C, 100 µm in D.

Previous studies demonstrate that flow-mediated remodeling is mediated by NADPH oxidase subunit p47 [32]. We evaluated the expression of p47 in WT and CaMKIIδ−/− right carotid arteries after injury. Whereas p47 expression increased on day 14 after ligation in WT mice, the p47 immunofluorescence was significantly lower in CaMKIIδ−/− samples at baseline and after ligation (Figure 3A). Accordingly, we detected a trend towards decreased ROS production in CaMKIIδ−/− carotid arteries (Figure 3B). In order to further test the role of CaMKII oxidation in arteriogenesis in our model, we used a new knock-in mouse model in which CaMKIIδ cannot be activated by oxidation due to mutation of Met 281,282 to Val (CaMKII MV). Oxidized CaMKII levels were increased in WT but not CaMKII MV right carotid arteries (Figure 3C, S4 in File S1). We detected a significant increase in IEL and EEL circumference in the right carotids from WT but not CaMKII MV mice on day 28 after ligation (Figure 3D and E). No compensatory increase in autophosphorylated CaMKII was seen in CaMKII MV mice (data not shown). These data suggest that oxidized CaMKII may be an important upstream signal for arteriogenesis.

### Macrophage-derived CaMKIIδ Expression is Increased in Arteriogenesis

**Figure 4:**
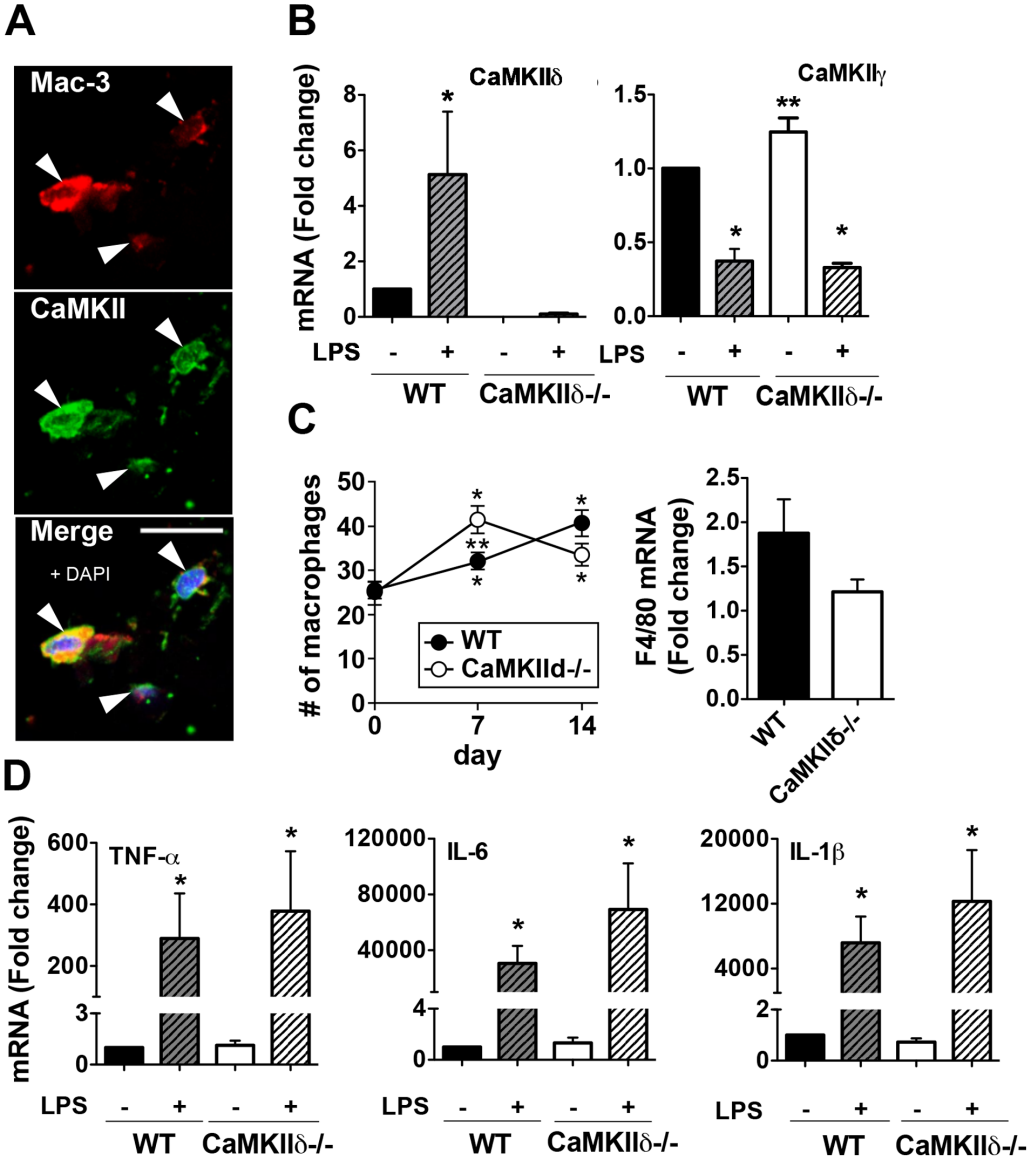
Adventitial macrophages express CaMKII. (A) Double labeling of adventitial macrophages from WT mice for total CaMKII (green) and the macrophage marker Mac-3 (red) on day 7 after ligation. Nuclei were stained with DAPI (blue). Arrowheads indicate macrophages. Scale bar = 30 µm. (B) Quantitative RT-PCR for CaMKIIδ and γ in WT and CaMKIIδ−/− BMMs before and after treatment with 1 µg/ml LPS for 6 hr. (C) Left panel, quantification of the number of Mac-3-labeled macrophages in sections of right WT and CaMKIIδ−/− carotid arteries after left carotid ligation. Right panel, quantitative RT-PCR for the macrophage marker F4/80 at day 7 post-ligation relative to baseline (p = 0.152 between genotypes). (D) Quantitative RT-PCR for IL-6, IL-1β and TNF-α in WT and CaMKIIδ−/− BMMs at baseline and 6hr after LPS treatment (1 µg/ml). (n = 9 mice per group) *p<0.05 compared to baseline, **p<0.05 compared to WT.

We next asked if the arteriogenesis-promoting activities of CaMKIIδ reside in a particular cell type. We focused on macrophages because macrophage depletion has been shown to prevent flow-mediated remodeling [4]. We found that CaMKII colocalized with macrophages in the adventitia of right carotid arteries as determined by anti-Mac-3 immunofluorescence (Figure 4A). Next, we used bone marrow-derived macrophages (BMMs) isolated from WT and CaMKIIδ−/− mice to confirm the presence and inducibility of CaMKII. Since toll-like receptor 4 (TLR4) activation contributes to arteriogenesis [34], [35], we assessed BMM CaMKIIγ and CaMKIIδ mRNA expression following exposure to lipopolysaccharide (LPS), a known TLR4 agonist. Low levels of both CaMKIIγ and CaMKIIδ were detected in WT BMMs at baseline, and exposure to LPS promoted a 5-fold increase in CaMKIIδ mRNA levels (Figure 4B, left panel). Interestingly, LPS exposure significantly decreased CaMKIIγ mRNA regardless of genotype (Figure 4B, right panel), suggesting that expression of CaMKIIγ and CaMKIIδ are regulated through different pathways. These findings further support the concept that the increased adventitial CaMKII expression in right WT carotid arteries (Figure 2C) is mainly due to increased expression of the CaMKIIδ isoform.

Arteriogenesis is a multi-step process, including macrophage infiltration, secretion of inflammatory cytokines [6], [8], [24], [32]–[35] and activation of matrix metalloproteinases (MMPs) [9], [10]. Thus, we evaluated whether CaMKIIδ deficiency alters macrophage infiltration associated with flow-mediated remodeling. The number of adventitial macrophages, as detected by Mac-3 immunostaining, was increased at 7 days post-ligation in both WT and CaMKIIδ−/− carotid arteries (left panel, Figure 4C). The greater number of macrophages in CaMKIIδ−/− mice 7 days post-ligation suggests that blunted arteriogenesis in CaMKIIδ−/− mice is not due to impaired monocyte/macrophage recruitment. In contrast, quantitative RT-PCR for F4/80 mRNA, a marker of mature macrophages, in lysates of carotid arteries on day 7 demonstrated a 1.8-fold in WT and a lesser 1.2-fold increase in CaMKIIδ−/− mice (right panel, Figure 4C). Similar results were seen by immunostaining for F4/80 (data not shown). These findings point towards a role for CaMKIIδ in macrophage maturation.

We analyzed whether other bone marrow-derived cells infiltrate the perivascular space in this model. At 7 days post-injury, we detected few lymphocytes and endothelial progenitor cells. Numerous granulocytes were identified in the perivascular space following injury, with a more pronounced increase with CaMKIIδ deficiency (Figure S5 in File S1).

We next investigated the expression of macrophage-derived cytokines that are enhanced in flow-mediated remodeling [35]. At baseline and after LPS exposure, IL-6, IL-1β, and TNF-α mRNA levels were similar in BMMs isolated from WT and CaMKIIδ−/− mice (Figure 4D), suggesting that CaMKII regulation of other macrophage-derived factors, for example MMPs, may mediate outward remodeling.

### MMP9 Expression is Reduced in CaMKIIδ−/− Carotid Arteries during Remodeling

**Figure 5:**
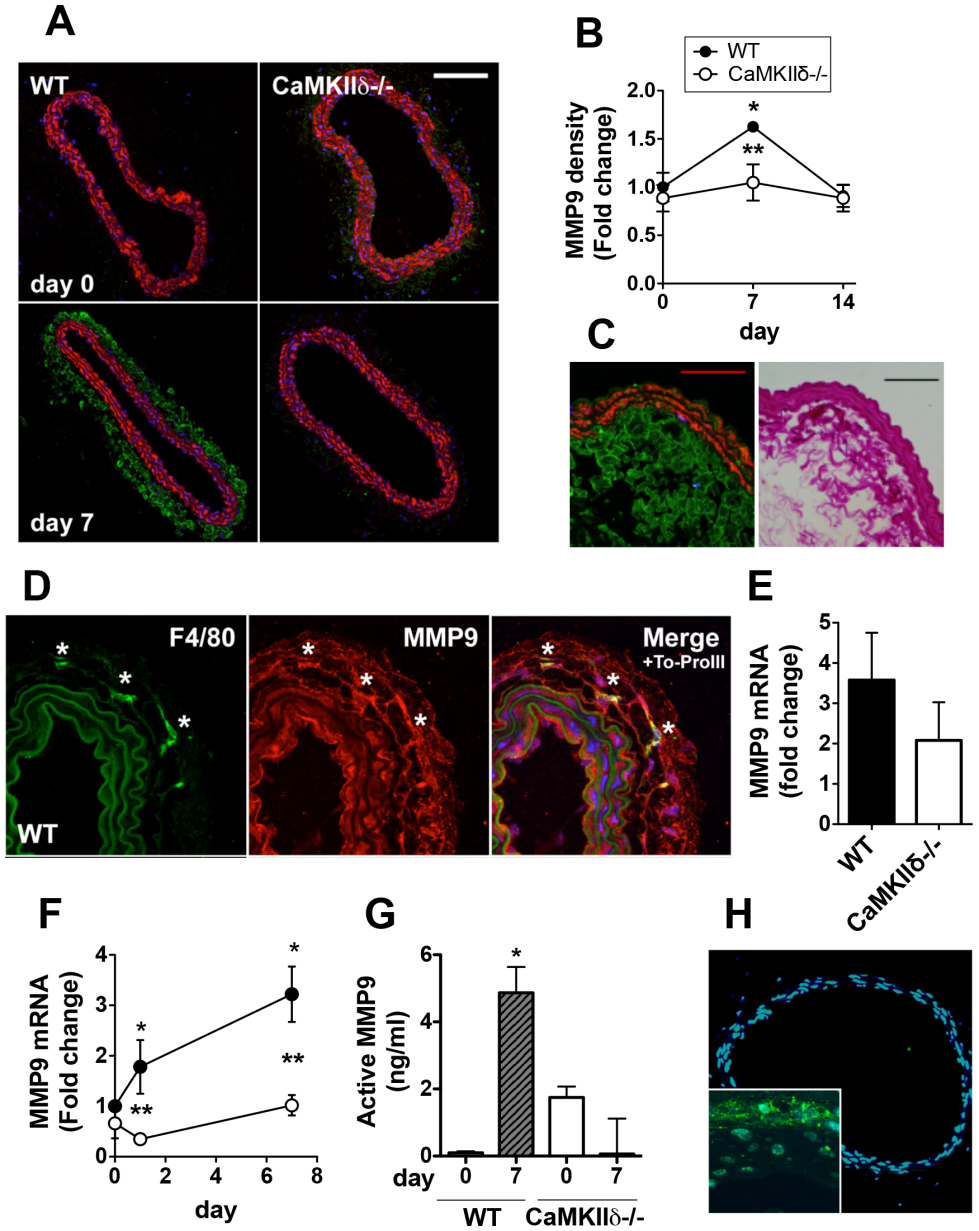
CaMKIIδ promotes MMP9 expression in flow-mediated remodeling. (A) MMP9 immunolabeling (green; SM-actin, red; To-ProIII, blue) in right carotid artery sections from WT and CaMKIIδ−/− mice before and 7 days after left carotid ligation. Scale bar = 100 µm. (B) Quantification of MMP9 staining intensity. (C) Magnification of MMP9 adventitial labeling (left panel, green; SM-actin, red) and Masson Trichrome staining (right panel) in WT right carotid artery section 7 days post-ligation. Scale bar = 50 µm. (D) Adventitial MMP9 (red) and macrophage marker F4/80 (green) double-labeling in right carotids from WT mice. Nuclei were stained with To-ProIII (blue); * indicates co-localization. (E) Quantitative RT-PCR for MMP9 in BMMs 6 hr after addition of LPS (1 µg/ml, p = 0.053). (F) Quantitative RT-PCR for MMP9 in right carotid arteries 1 and 7 days after left ligation. (G) MMP9 activity in right carotid artery homogenates at baseline and 7 days after ligation. *p<0.05 compared to day 0; **p<0.05 compared to WT.

Increased activity of MMPs, particularly MMP9, is known to promote arteriogenesis [9], [10]. Recent evidence by our group and others has identified CaMKII as a regulator of MMP9 [17], [30], [36]. We therefore examined MMP9 expression post-ligation and detected a significant increase in adventitial MMP9 immunostaining in WT but not in CaMKIIδ−/− carotid arteries (Figure 5A, B). On day 7, MMP9 co-localized with both the adventitial extracellular matrix (Figure 5C) and macrophages (Figure 5D), suggesting that macrophages are likely an important source of MMP9 that is then secreted into the extracellular space and activated during arteriogenesis. Accordingly, a 45% decrease in MMP9 expression was also observed in isolated BMMs from CaMKIIδ−/− mice following LPS exposure (Figure 5E). Our investigation of MMP9 expression in homogenized right carotid arteries revealed a significant increase in MMP9 mRNA in WT mice on days 1 and 7 following ligation, similar to other published evidence [9], while this increase was not observed in CaMKIIδ−/− carotids (Figure 5F). MMP9 activity on day 7 increased significantly over baseline in homogenized WT but not CaMKIIδ−/− carotid arteries (Figure 5G). Thus, our data strongly suggest that CaMKIIδ-dependent induction of MMP9 is an early event in arteriogenesis.

CaMKII has been shown to regulate gene transcription via phosphorylation of histone deacetylases 4/5 (HDAC4/5), which relieves repression of the transcription factor myocyte enhancer factor-2 (MEF-2) [13]. Given that MEF-2 is expressed by macrophages [37], [38] and induces transcription of other MMP family members [38], [39], we examined whether one mechanism by which CaMKII promotes MMP9 expression in arteriogenesis is via increased MEF2 transcriptional activity. For these studies, we crossed CaMKIIδ−/−mice with MEF2 reporter mice that contain LacZ downstream of three MEF2 promoter binding sites [17], [40]. Following left carotid ligation, we did not observe MEF2 transcriptional activity in the right carotids of control MEF2 reporter mice regardless of CaMKIIδ expression (Figure 5H), suggesting other factors contribute to CaMKII regulation of MMP9 expression in arteriogenesis.

### Transplantation of WT Bone Marrow into CaMKIIδ−/− Mice Recovers Remodeling

**Figure 6:**
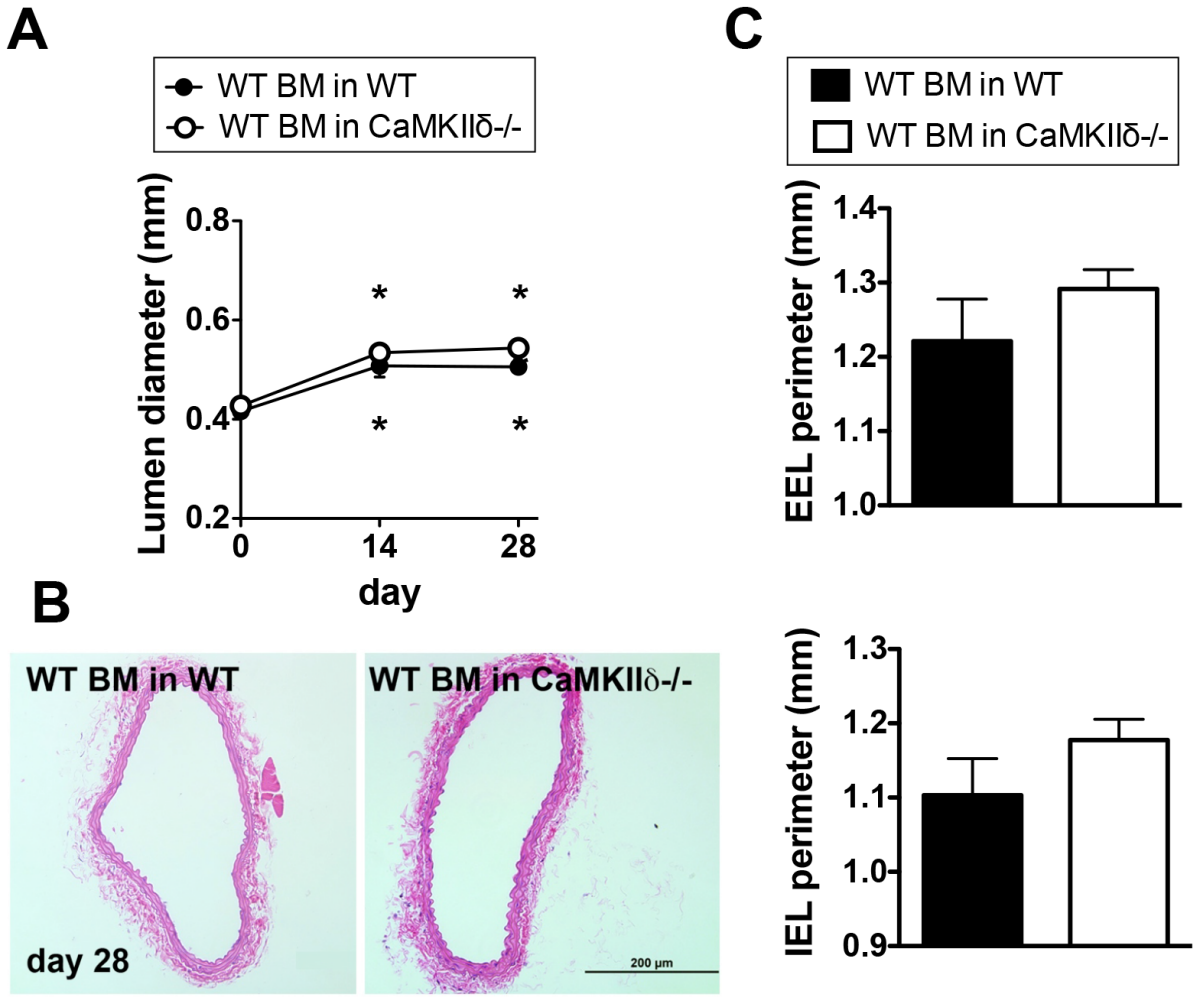
Transplantation of WT bone marrow cells into CaMKIIδ−/− mice restores arteriogenesis. (A) Quantification of the anterior-posterior diameter of right carotid arteries of WT or CaMKIIδ−/− mice transplanted with WT BM (n = 6 for WT and n = 10 for CaMKIIδ−/− mice, *p<0.05 compared to day 0). (B) Representative H&E-stained WT and CaMKIIδ−/− right carotid arteries after transplantation of WT bone marrow. Carotid artery ligations were performed 8 weeks after BM transplantation. Scale bar = 200 µm (C) Quantification of the perimeter of the IEL and EEL (n = 6 for WT and n = 10 for CaMKIIδ−/− mice).

Given that one current therapeutic strategy for occlusive vascular disease is bone marrow transplantation, we tested whether transplantation of WT bone marrow restores arteriogenesis in CaMKIIδ−/− mice. We first transplanted WT mice with WT bone marrow and performed carotid ligations 12 weeks after transplantation. On day 14 and 28 post-ligation, we detected significantly increased *in vivo* carotid diameter by ultrasound (Figure 6A), similar to our initial results in WT mice (Figure 1E). In addition, the IEL and EEL perimeters were significantly increased on day 28 (Figure 6B, C). Transplantation of WT bone marrow into CaMKIIδ−/− mice resulted in an increased carotid diameter (Figure 6A), with perimeters similar to those in WT mice (Figure 6B, C). These data demonstrate that transplantation of WT bone marrow normalizes and completely restores arteriogenesis in CaMKIIδ−/− mice.

### Blunted Arteriogenesis in CaMKIIδ−/− Mice is not due to Differences in Endothelial Adhesion Molecule Expression or VSMC Proliferation

**Figure 7:**
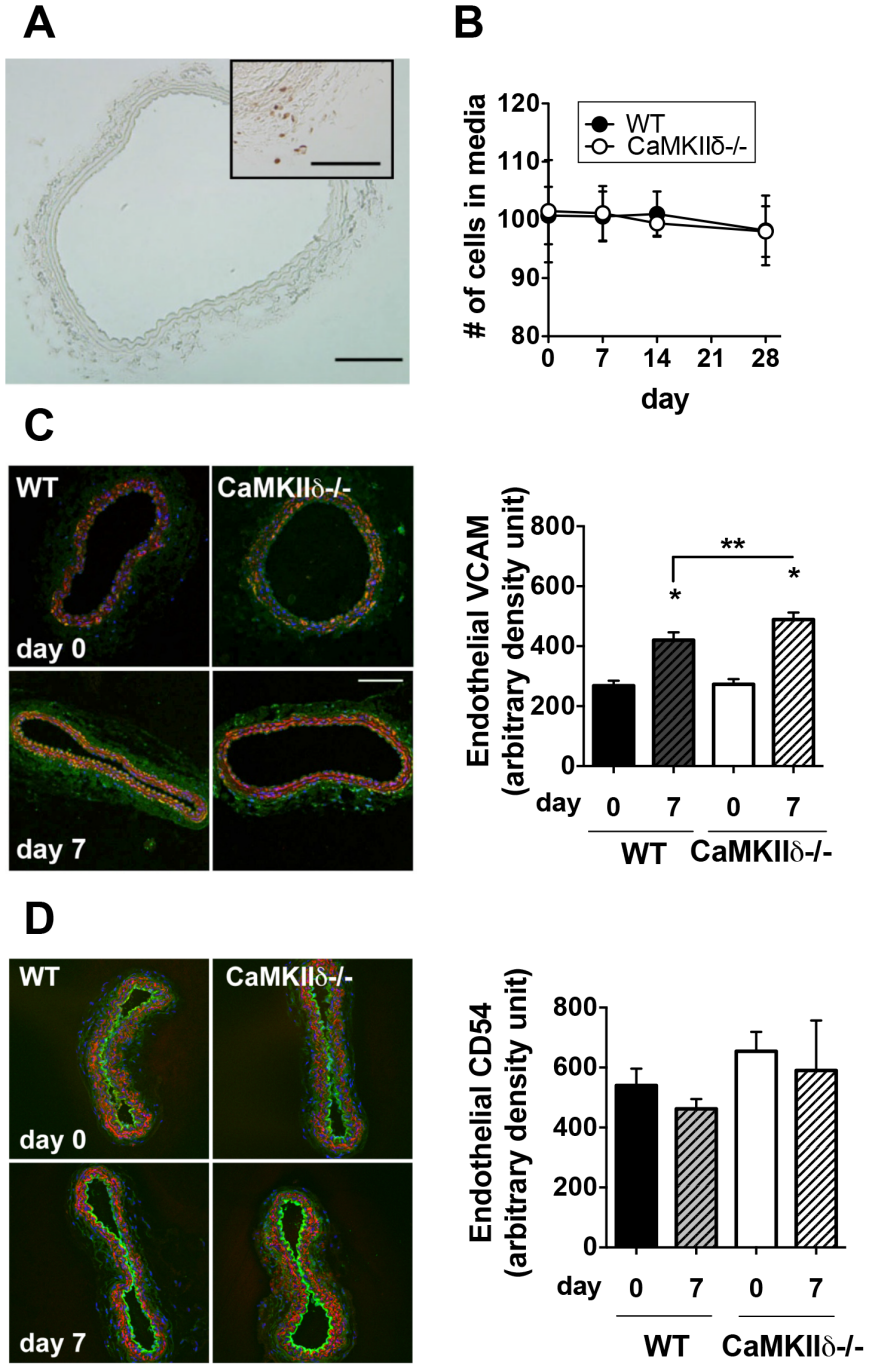
CaMKIIδ deletion does not decrease VSMC proliferation or endothelial expression of VCAM-1 or CD54 (ICAM-1). (A) Representative image of BrdU-labeling of WT right common carotid artery 28 days post-ligation. Inset: neointima in the left carotid artery of the same mouse as positive control. Scale bar = 100 µm. (B) Quantification of medial cells from right carotid artery sections labeled for SM-actin and nuclei (To-ProIII). (C) Left panels, representative immunofluorescent images of VCAM-1 (green) in the right common carotid artery 7 days post-ligation (SM-actin, red; nuclei, blue). Scale bar = 100 µm. Right panel, quantification of endothelial VCAM-1 labeling. *p<0.05 compared to baseline; **p<0.05 compared to WT. (D) left panels, representative immunofluorescent images of CD45 (ICAM-1) (green) in the right common carotid artery 7 days post-ligation (SM-actin, red; nuclei, blue). Right panel, quantification of endothelial CD45 labeling (n = 5).

Flow-mediated remodeling results in an increase in the number of vascular smooth muscle cells (VSMC) [41], [42]. We have previously established that CaMKIIδ mediates VSMC proliferation [16]. However, proliferation as assessed by BrdU incorporation was not increased in WT or CaMKIIδ−/− right carotid arteries post-ligation (Figure 7A). In addition, the number of cells in the media was similar between WT and CaMKIIδ−/− arteries (Figure 7B).

Next, we investigated whether CaMKIIδ regulates the expression of the endothelial adhesion molecule, vascular cell adhesion molecule 1 (VCAM-1), which is up-regulated in models of flow-mediated remodeling [6], [8]. We found higher levels of VCAM-1 with increased flow (Figure 7C). However, in contrast to our hypothesis, we observed a greater increase in CaMKIIδ−/− carotid arteries. Several studies have reported a role for CD54 (ICAM-1) in increasing vascular permeability [6]. Thus, we tested the effect of CaMKIIδ deletion on CD54 expression and did not detect a significant difference (Figure 7D). Similarly, no differences in MCP-1 expression were observed (data not shown). These data do not support that CaMKII promotes arteriogenesis by actions on adhesion molecules.

## Discussion

Arteriogenesis is largely a compensatory response to a sustained increase in blood flow and shear stress on the vascular wall. Several key findings in our study identify CaMKII as an important regulator of arteriogenesis. 1) Under high flow conditions, CaMKII is strongly expressed, especially in the endothelium and adventitia, and is activated by oxidation. Deletion of CaMKIIδ abolishes outward remodeling, revealing a pivotal role for CaMKIIδ in arteriogenesis. 2) Using a knock-in model in which oxidative CaMKII activation is abrogated by point mutations of methionine residues in the autoregulatory domain, we provide evidence that oxidative activation of CaMKII is not a by-stander effect but is causally linked to CaMKII function in vascular remodeling. These findings also identify CaMKII as a novel target and mediator of ROS-dependent signaling in the vasculature. 3) Our data demonstrate that CaMKII is expressed in macrophages that infiltrate the vascular wall and controls the outward remodeling process, likely through regulation of MMP9 expression. 4) Transplantation of WT bone marrow into CaMKIIδ−/− mice normalizes flow-mediated remodeling. Taken together, our data identify CaMKII activation as a potential target to induce arteriogenesis.

Oxidative activation of CaMKII has been described recently in myocardial pathology and linked to increased myocardial rupture after infarction [30] and sinus node dysfunction [43]. We recently reported that oxidized CaMKII is present in the neointima after vascular injury [17].

One of the major findings of the present study is the increase in oxidized but not phosphorylated CaMKII during flow-mediated remodeling. We and others have shown that NADPH oxidase subunit p47-dependent ROS are increased in response to flow-mediated remodeling [31], which likely contributes to the CaMKII oxidative activation observed in our study. A surprising and novel aspect of our study is the decrease of p47 and ROS in CaMKIIδ-deficient carotids both at baseline and following injury. Regulation of NAPDH oxidase 5 by CaMKII has been proposed in the literature previously [44].

CaMKII is activated in human peripheral blood mononuclear cells in response to LPS (14); however, its function in macrophages has not been extensively studied. Mishra and colleagues reported that the macrophage migration mediated by CD44 in response to LPS is independent of CaMKII [45], a finding that is in agreement with our data that revealed a greater number of macrophages in the carotid wall in CaMKIIδ−/− mice. Tang and colleagues demonstrated modest increases in IL-1β, TNF-α and IL-6 mRNA levels in the right carotid artery in a similar model. The cytokine production was attributed to the intrinsic vascular wall cells [46] rather than the infiltrating macrophages. In a recent study in peritoneal macrophages [15], knockdown of CaMKIIα resulted in decreased expression of IL-6 and TNF-α in response to LPS at similar time points. In our studies, we did not detect any significant difference in the same cytokines between WT and CaMKIIδ−/− macrophages, which may be due to a different macrophage isolation protocol or isoform-specific action of CaMKII on gene transcription. In contrast to the significantly greater increase in the number of infiltrating macrophages in CaMKIIδ−/− arteries by day 7 post-ligation, we noted that F4/80, a marker of mature stages of macrophage differentiation [47], only weakly increases (right panel, Figure 4C). This finding suggests that CaMKIIδ may have a role in macrophage differentiation that will warrant further investigation.

The function of CaMKII in the endothelium is widely unknown. Few reports have concentrated on this topic. It is currently assumed that CaMKII mediates endothelial nitric oxide synthase activation, actin reorganization and endothelial barrier dysfunction [22], [23]. Based on these data and the strong expression and ox-CaMKII labeling in the endothelium of WT mice under high flow conditions, we hypothesized that CaMKIIδ deletion decreases endothelial permeability, thus resulting in decreased monocyte infiltration and remodeling of the vascular wall. However, the number of macrophages in the vascular wall was not reduced in CaMKIIδ−/− mice, but rather increased compared to WT (left panel, Figure 4C). In addition, we tested whether endothelial CaMKII regulates adhesiveness through VCAM-1, ICAM-1, and MCP-1 expression, based on a report in tracheal smooth muscle cells [48]. Under high flow conditions, we did not detect a decrease in expression of these adhesion molecules in the endothelium.

MMP inhibition or knock out of MMP9 reduces flow-mediated remodeling in this arteriogenesis model [9]. We detected MMP9 protein expression under high flow mainly in the endothelium and adventitia on day 7 after ligation, in contrast to the previous study that reported significant MMP9 labeling in medial VSM cells. This difference may be explained by the difference in time points chosen (day 3 vs. day 7). MMP9 expression in macrophages is induced by IL-6. Since we did not detect any difference in LPS-induced IL-6 expression in macrophages, the observed difference in MMP9 expression points towards a CaMKII-specific effect on MMP9 mRNA expression or stability. We recently described that MMP9 mRNA stability is decreased in response to CaMKIIδ deletion in VSMC [17]. In models of flow-mediated remodeling, ROS interacts with nitric oxide to form peroxynitrite [32], [33], which in turn activates MMP9 and facilitates outward remodeling. Here, we present evidence that CaMKII is directly activated by peroxynitrite through oxidation. Thus, we propose that increased peroxynitrite production in models of arteriogenesis activates CaMKII that contributes to structural remodeling by regulating MMP9.

Bone marrow transplantation has received considerable attention as an experimental treatment option in occlusive vascular disease. This study underlines the importance of bone marrow-derived cells for arteriogenesis. Our interpretation that macrophages are the main drivers of flow-mediated outward remodeling is supported by numerous studies that used pharmacological macrophage depletion to demonstrate inhibition of the remodeling process [4], [7], [49]. While bone marrow-derived endothelial progenitor cells have been postulated to incorporate into the endothelium in the past [50], previously published data suggest that these cells are leukocytic infiltrates in the perivascular space and secrete arteriogenic substances. In our experiments, we detected few infiltrating endothelial progenitor cells. In contrast, granulocyte infiltration in the right carotid perivascular space was increased following left carotid injury, especially in CaMKIIδ−/− arteries. This finding correlates with the greater number of macrophages in the right CaMKIIδ−/− arteries after left ligation. These data are suggestive of a potential mechanism for the increased macrophage recruitment as has been previously described [51].

In summary, this study provides *in vivo* and *in vitro* evidence that CaMKIIδ controls flow-mediated outward remodeling. Moreover, we demonstrated that oxidative activation of CaMKII mediates the remodeling process. Our findings reveal that CaMKIIδ expression is dynamically regulated in endothelium and adventitial monocytes and macrophages, the latter being the source of CaMKIIδ controlling the remodeling process. Our data also provide evidence that CaMKII regulates MMP9 expression in macrophages, which we believe contributes to the observed phenotype. These data provide novel insights into the mechanisms involved in arteriogenesis and raise questions regarding whether CaMKII modulation in clinical settings could increase collateral formation in occlusive arterial disease.

## Supporting Information

File S1 Supplemental Figures. Figure S1, Velocity time integral and blood pressure in WT and CaMKIIδ−/− mice; Figure S2, Densitometry of CaMKII and ox-CaMKII in WT and CaMKIIδ−/− mice; Figure S3, Peroxynitrite activates CaMKII; Figure S4, ox-CaMKII and CaMKII in WT and CaMKII MV mice; Figure S5, BM-derived cells in the perivascular space: endothelial derived stem cells, lymphocytes and granulocytes.
(PDF)
