# Supplementary material for: The Multifunctional Ca2+/Calmodulin-Dependent Kinase IIδ (CaMKIIδ) Regulates Arteriogenesis in a Mouse Model of Flow-Mediated Remodeling
Source: PLoS One. 2013 Aug 8;8(8):e71550. doi: 10.1371/journal.pone.0071550 (PMC3738514; doi:10.1371/journal.pone.0071550)

SUPPLEMENTAL FIGURE LEGENDS

Supplemental Figure S1: Velocity time integral and blood pressure in WT and CaMKIIδ-/- mice.

(A) The blood pressure in WT and CaMKIIδ-/- mice was measured by tail cuff method on day 13 and 27 after a 7-day training period. Midazolam was administered as used for the ultrasound experiments that were performed the following day. No significant difference between the genotypes was detected (n= 10 per time point, measurements on day 13 and 27 are combined by genotype).

(B) The velocity time interval was determined on day 0, 14 and 28 by pulsed Doppler interrogation in 10 age- and sex-matched WT and 10 CaMKIIδ-/- mice, demonstrating an increase in VTI in both genotypes. During the same examination, 2-D cross-sectional images of the carotid arteries were obtained as displayed in Figure 1D.

Supplemental Figure S2: Densitometry of CaMKII and ox-CaMKII in WT and CaMKIIδ-/- mice

(A) Immunofluorescent labeling for CaMKII was quantified in endothelium and adventitia of right carotid arteries at baseline and on day 7, 14 and 28 after left carotid ligation. The density was determined using Image J software and displayed as fold increase over the density at baseline for each genotype (n=9 per group, * $p<0.05$ compared to baseline, ** $p<0.05$ compared to WT). The labeling in the media was low and not different between time points and genotypes. Representative images are also shown in Figure 2B.

(B) The density of ox-CaMKII staining was determined as described in (A). Representative images are also shown in Figure 2C.

Supplemental Figure S3: Peroxynitrite activates CaMKII.

CaMKIIδ (GenBank #NP_001020609) was generated using the Bac-to-Bac baculovirus system (Invitrogen) and purified on a calmodulin-agarose column. Mutant CaMKII MV cDNAs was generated using a QuikChange site-directed mutagenesis kit (Stratagene). For CaMKII activity assays, purified CaMKII was pretreated with 200 μM $CaCl_2$ and 1 μM CaM on ice for 1 min. The protein was then exposed to peroxynitrite or vehicle at the described concentrations for 10 min. CaMKII activity was measured as a function of ^{32}P-ATP incorporation into a synthetic substrate (syntide-2) at 30°C. (* $p<0.05$ compared to baseline, ** $p<0.05$ compared to WT).

Supplemental Figure S4: ox-CaMKII and CaMKII in WT and CaMKII MV mice. (A) Densitometry of endothelial and adventitial ox-CaMKII labeling in the right carotid artery of WT and CaMKII MV mice (MV) at baseline (day 0) and 14 days after left carotid ligation. Only faint staining was seen in the media.

(B) Representative images of immunolabeling for total CaMKII (green; SM–actin red; blue nuclei) demonstrate increased presence of CaMKII in the endothelium of WT carotid arteries on day 14 after ligation which was significantly decreased in CaMKII MV mice. The same trends were seen in the adventitia.

Supplemental Figure S5: BM-derived cells in the perivascular space: endothelial derived stem cells, lymphocytes and granulocytes. (A) Representative figure of CD31 (PECAM) staining in the WT carotid artery 7 days after left carotid ligation. Positive control: endothelium (60X, B), (C) Negative control without primary antibody. Only few, isolated cells in the perivascular space are CD31-positive, indicated by arrow (60X, D).

(E) Representative figure of CD3 staining in a WT carotid artery 7 days after left carotid ligation (20X, CD3 red, nuclear counterstain with Syto16 green). Spleen as positive

control (60X, F), (G) negative control without primary antibody (20X). Only very few, isolated cells in the perivascular space were CD3-positive, indicated by arrow (60X, H). (I) Representative figure of CD177 staining in a WT carotid artery 7 days after left carotid ligation (20X, inset (L) 60X). (J) Positive control spleen (60X), (K) negative control without primary antibody (20X). Numerous cells in the perivascular space were CD177-positive (L). (M) Quantification of CD177-positive cells in the perivascular space per section. (n=5 per group, * $p<0.05$ compared to baseline, ** $p<0.05$ compared to WT).

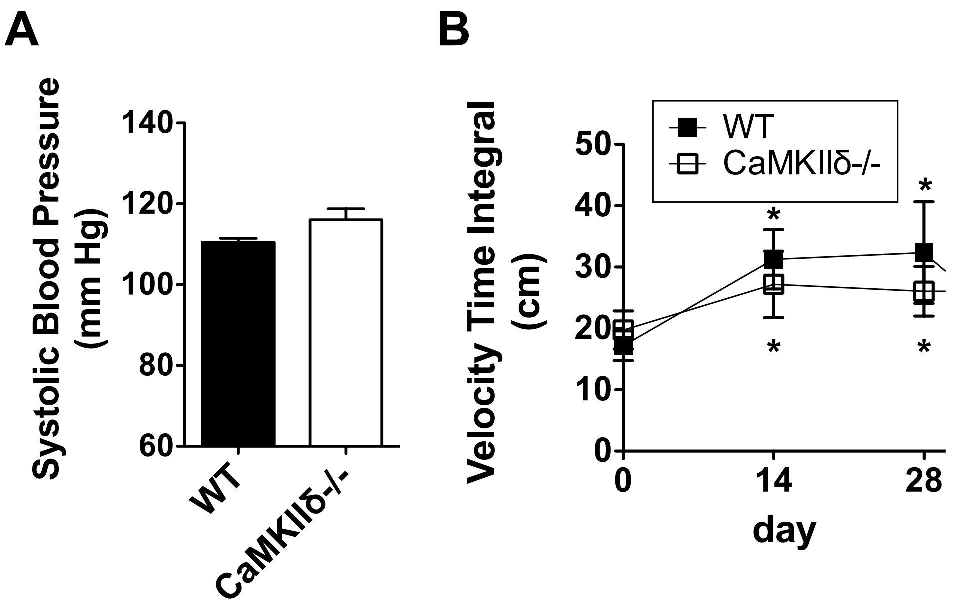
A
Systolic Blood Pressure
(mm Hg)
140
120
100
80
60
WT
CaMKIIδ-/-
B
Velocity Time Integral
(cm)
50
40
30
20
10
0
0
14
28
day
WT
CaMKIIδ-/-
*
*
*
*

CaMKIIδ controls flow-mediated outward remodeling Scott et al.
Supplemental Figure S2

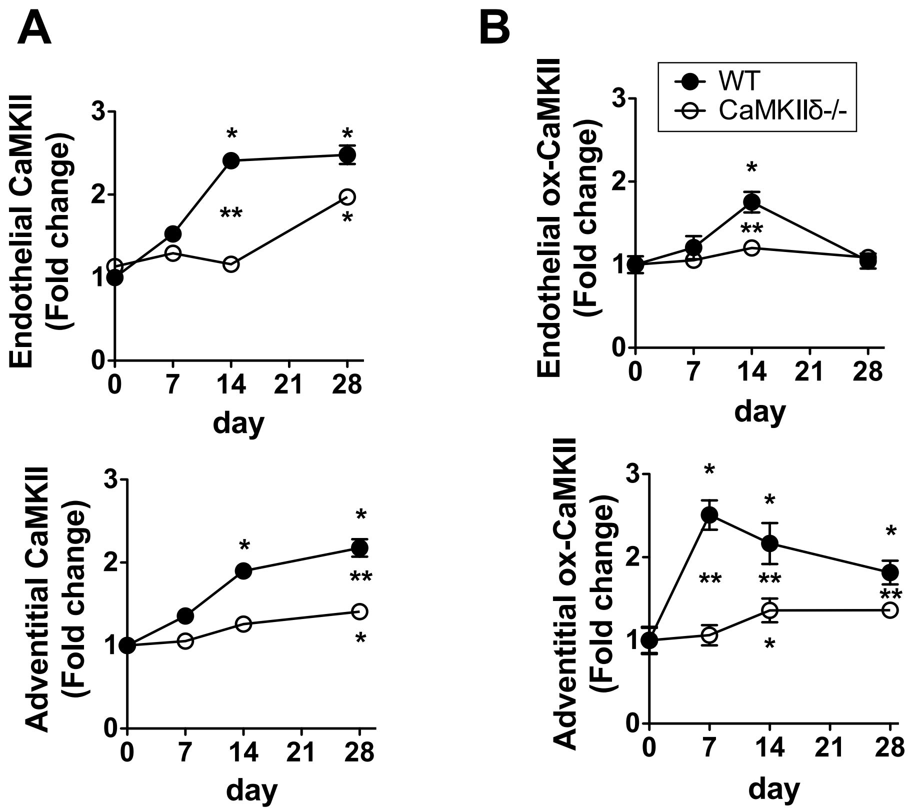

CaMKIIδ controls flow-mediated outward remodeling Scott et al.
Supplemental Figure S3

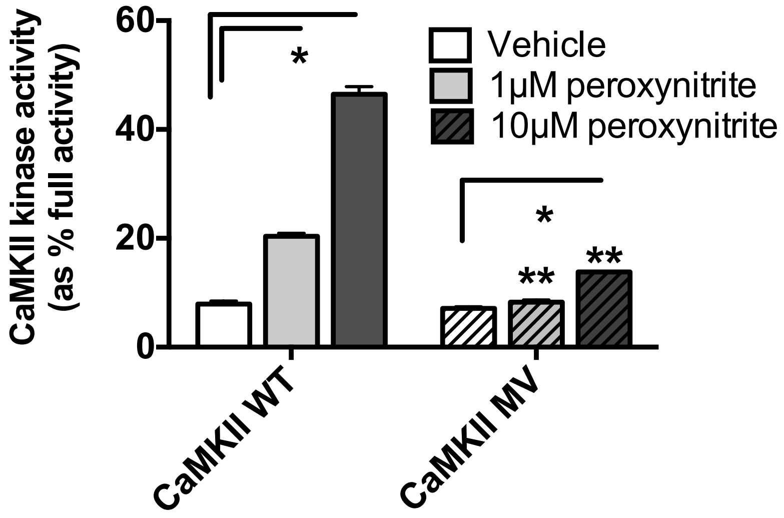

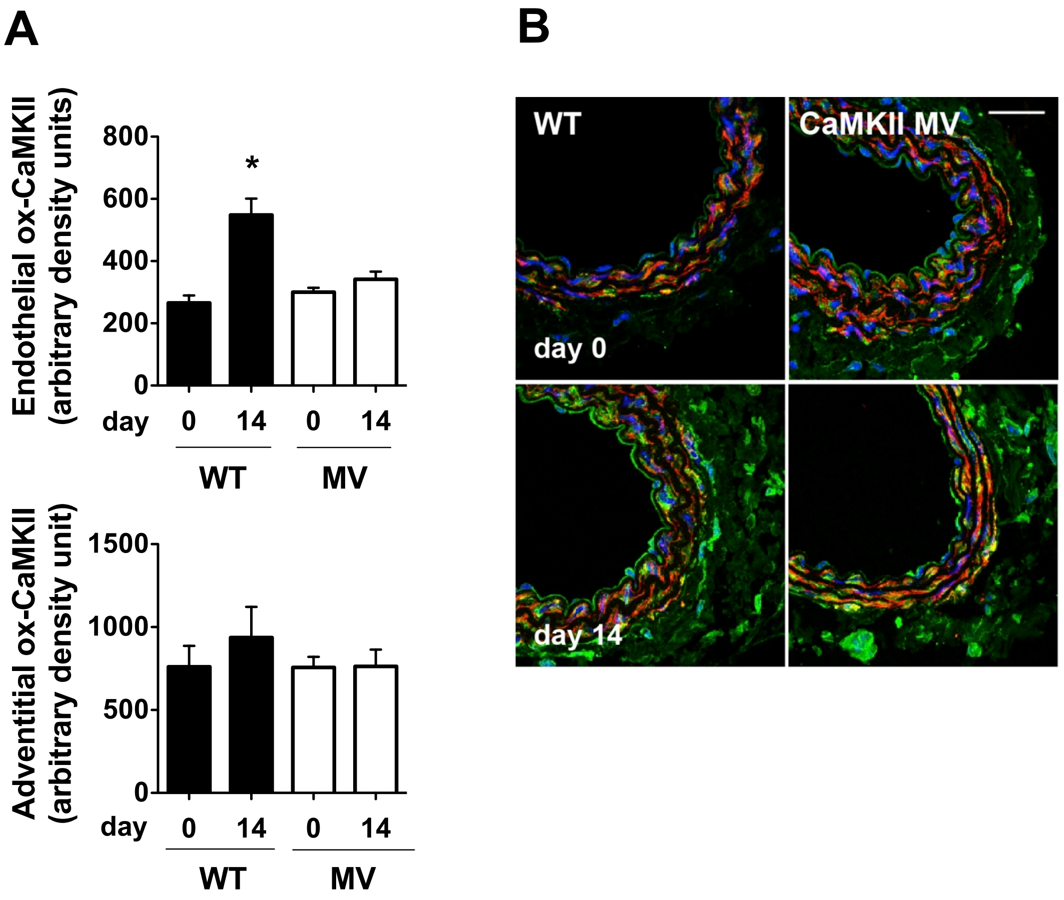
A
Endothelial ox-CaMKII (arbitrary density units)
800
600
400
200
0
*
day 0 14 0 14
WT MV
Adventitial ox-CaMKII (arbitrary density unit)
1500
1000
500
0
day 0 14 0 14
WT MV
B
WT
CaMKII MV
day 0
day 14

CaMKIIδ controls flow-mediated outward remodeling Scott et al.
Supplemental Figure S5

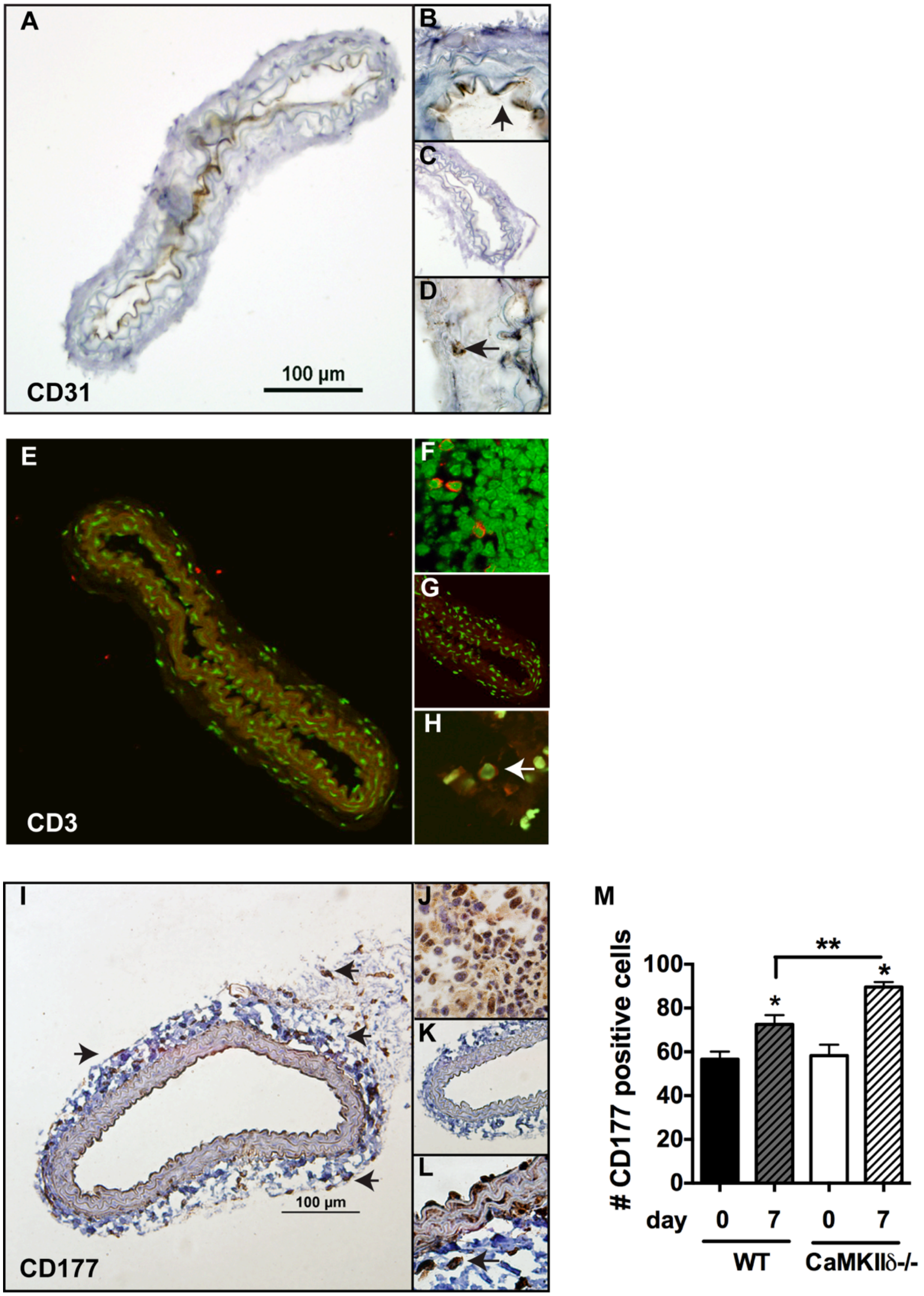

Supplement: File S1 — Supplemental Figures. Figure S1, Velocity time integral and blood pressure in WT and CaMKIIδ−/− mice; Figure S2, Densitometry of CaMKII and ox-CaMKII in WT and CaMKIIδ−/− mice; Figure S3, Peroxynitrite activates CaMKII; Figure S4, ox-CaMKII and CaMKII in WT and CaMKII MV mice; Figure S5, BM-derived cells in the perivascular space: endothelial derived stem cells, lymphocytes and granulocytes. (PDF) [file pone.0071550.s001.pdf]
